# Supplementary material for: Combination of T cell-redirecting bispecific antibody ERY974 and chemotherapy reciprocally enhances efficacy against non-inflamed tumours
Source: Nat Commun. 2022 Sep 7;13:5265. doi: 10.1038/s41467-022-32952-3 (PMC9452528; doi:10.1038/s41467-022-32952-3)
Supplement: Supplementary file 1 — Supplementary Information [file 41467_2022_32952_MOESM1_ESM.pdf]

Supplementary information

Combination of T cell-redirecting bispecific antibody ERY974 and chemotherapy reciprocally enhances efficacy against non-inflamed tumours

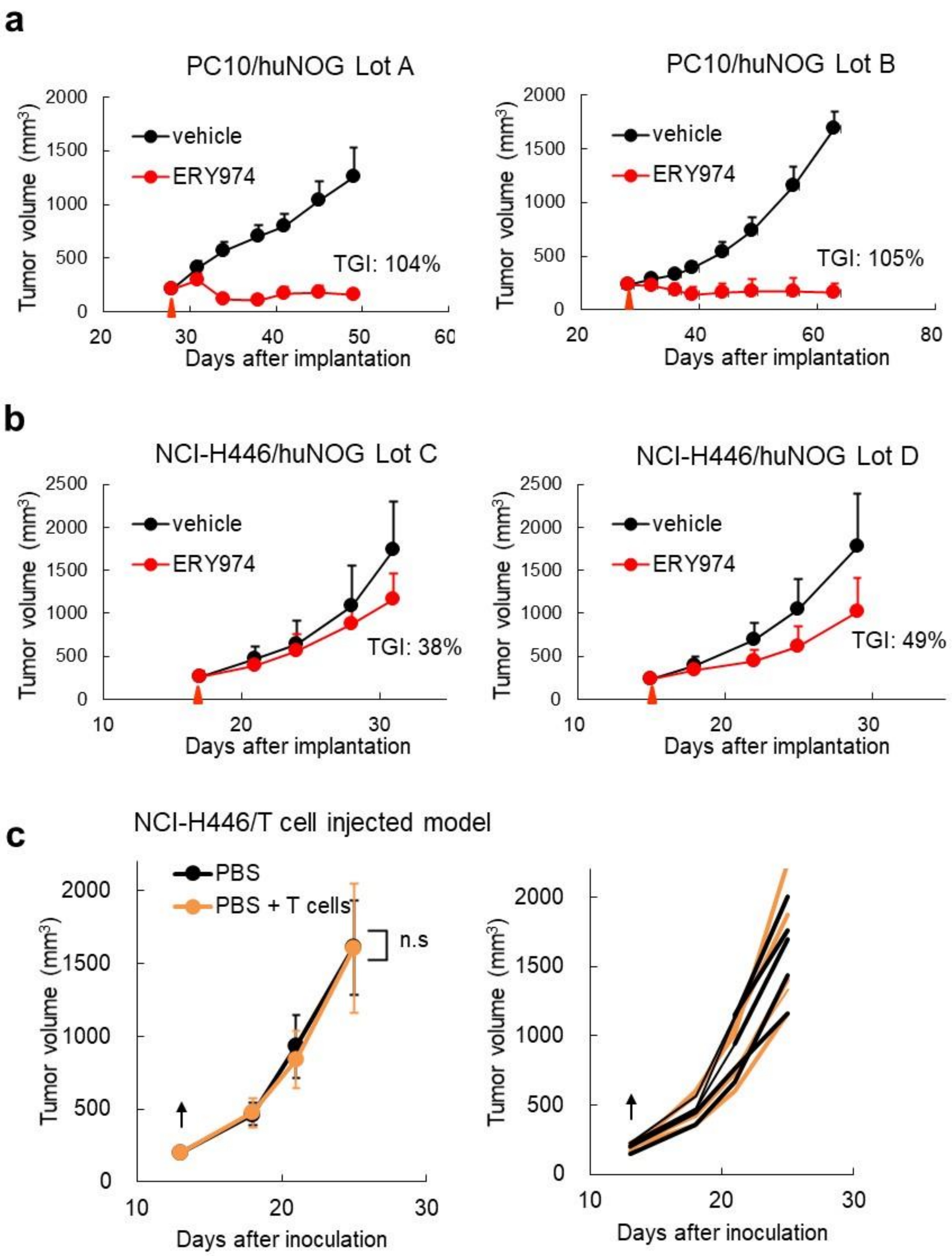

Supplementary Fig. 1. Efficacy of ERY974 in PC10 and NCI-H446 is not affected by different lots of huNOG. a, Antitumour efficacy of ERY974 in PC10 in huNOG lot A (left) (n = 5) and B (right) (n = 5, one mouse in the ERY974 group

was sacrificed due to body weight loss on day 44). ERY974 (1 mg/kg) was administered at the time denoted by the red arrow. Tumour volumes are presented as the mean ± SD. TGI at the final measurement point is shown in each figure. **b**, Antitumour efficacy of ERY974 in NCI-H446 in huNOG lot C (left) (n = 4, one mouse in vehicle group was sacrificed due to body weight loss on day 28) and D (right) (n = 5). ERY974 (1 mg/kg) was administered at the time denoted by the red arrow. Tumour volumes are presented as the mean ± SD. TGI at the final measurement point is shown in each figure. **c,** NCI-H446 tumour growth is not affected by allogenic T cells. NCH-H446 cells were inoculated into NOD/SCID mice with or without *ex-vivo* expanded T cells. The mean tumour size ± SD (n = 5) is shown on the left, and individual tumour size is shown on the right. Black lines indicate tumour size of mice treated with PBS, and orange lines indicate tumour size of mice treated with PBS plus *ex-vivo* expanded T cells (3 × 10^7 cells). The black arrow indicates the timing of T cell administration. Statistical analysis was conducted with Wilcoxon chi-square test. n.s. denotes no significance.

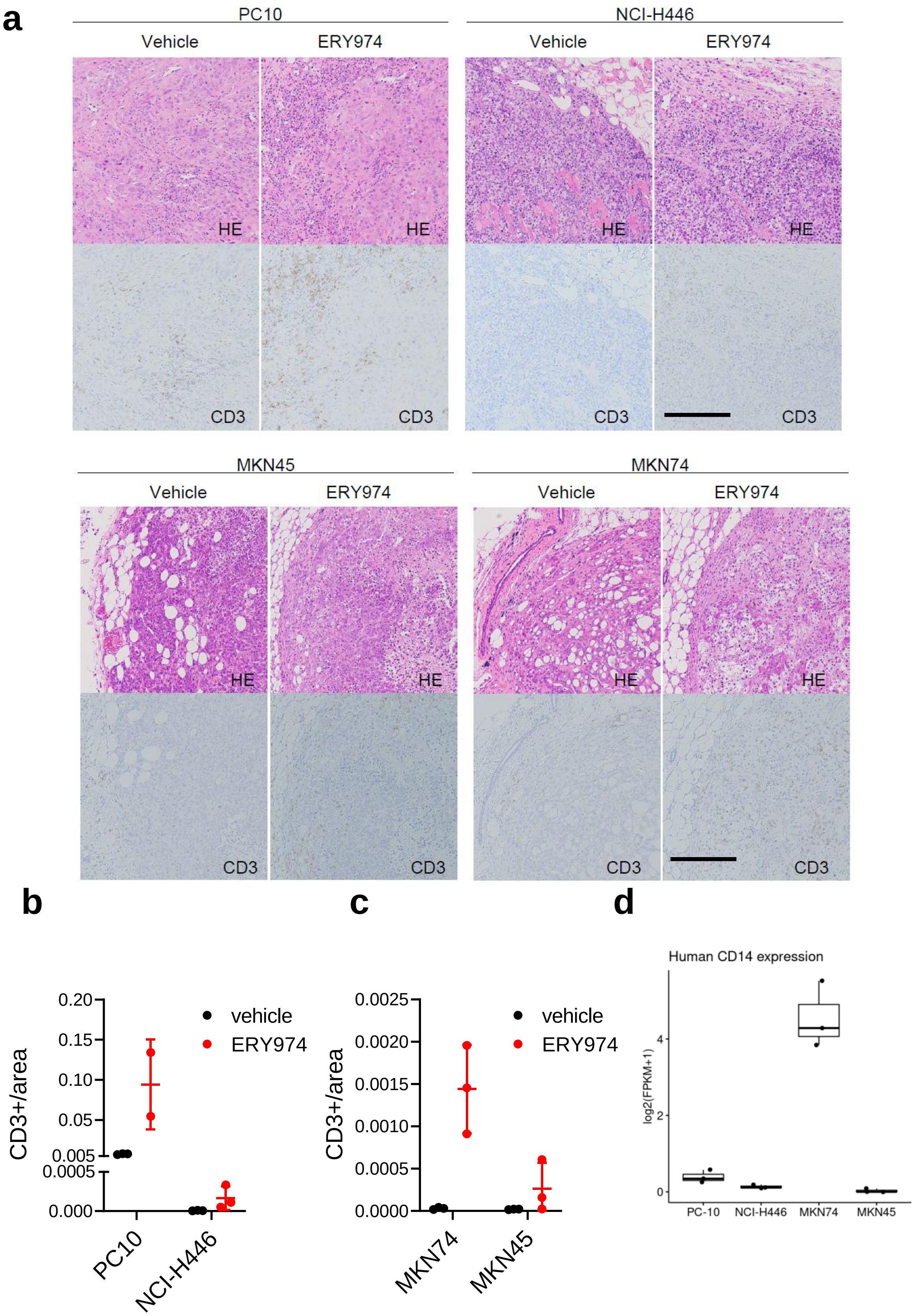
a
PC10
NCI-H446
Vehicle
ERY974
Vehicle
ERY974
HE
HE
HE
HE
CD3
CD3
CD3
CD3
MKN45
MKN74
Vehicle
ERY974
Vehicle
ERY974
HE
HE
HE
HE
CD3
CD3
CD3
CD3
b
c
d
CD3+/area
0.20
0.15
0.10
0.05
0.005
0.0005
0.000
vehicle
ERY974
PC10
NCI-H446
CD3+/area
0.0025
0.0020
0.0015
0.0010
0.0005
0.0000
vehicle
ERY974
MKN74
MKN45
Human CD14 expression
log2(FPKM+1)
4
2
0
PC-10
NCI-H446
MKN74
MKN45

e

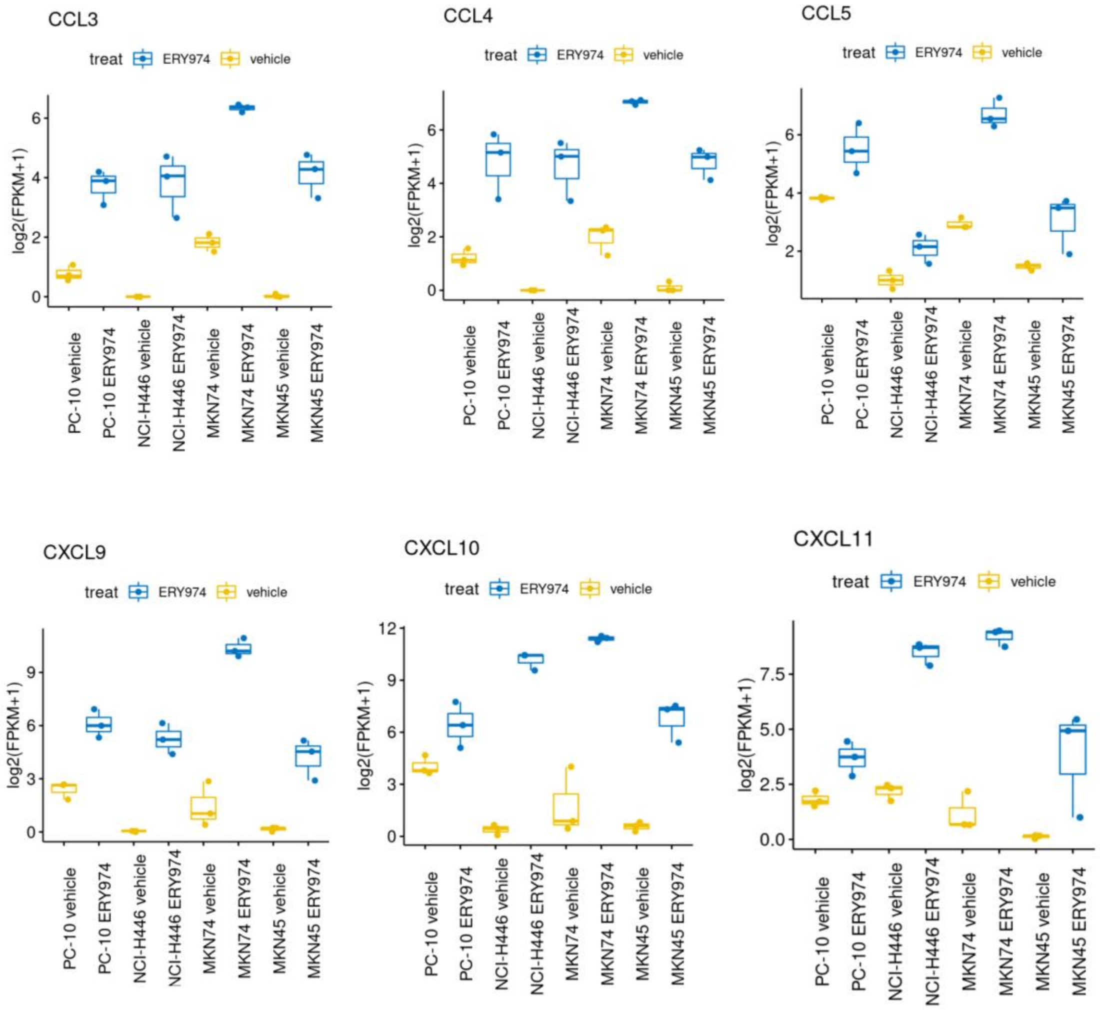

Supplementary Fig. 2. Efficacy of ERY974 is affected by the number of tumour-infiltrating immune cells at baseline in xenograft tumours of PC10, NCI-H446, MKN74, and MKN45 inoculated in huNOG mice. a. HE staining and hCD3 IHC of xenograft tumours (PC10, NCI-H446, MKN74, and MKN45). Tumours of PC10, NCI-H446, MKN74, and MKN45 inoculated in huNOG mice (n

= 3) were collected three days after vehicle-, or ERY974-treatment. Representative images are shown for each tumour. Scale bar is 500 μm **b.** Quantification of CD3-positive cells per area for PC10 and NCI-H446 tumours (GPC3-high) in **a**. Data are presented as the mean ± SD. **c.** Quantification of CD3-positive cells per area for MKN74 and MKN45 tumours (GPC3-low) in **a**. Data are presented as the mean ± SD. **d.** Quantification of human CD14 mRNA at baseline. Total RNA was extracted from PC10, NCI-H446, MKN74, and MKN45 tumours xenografted in huNOG (n = 3), and RNAseq was conducted. FPKM +1 score of human *CD14* was shown as log2 value. In the boxplot, centre lines show median values, box limits show upper and lower quartiles, and whiskers show minimum and maximum values. **e**. Quantification of human *CCL3, CCL4, CCL5, CXCL9, CXCL10*, and *CXCL11* mRNA. RNAseq was conducted as described above (n = 3). FPKM +1 score of human *CCL3, CCL4, CCL5, CXCL9, CXCL10, and CXCL11* was shown as log2 value. In the boxplot, centre lines show median values, box limits show upper and lower quartiles, and whiskers show minimum and maximum values.

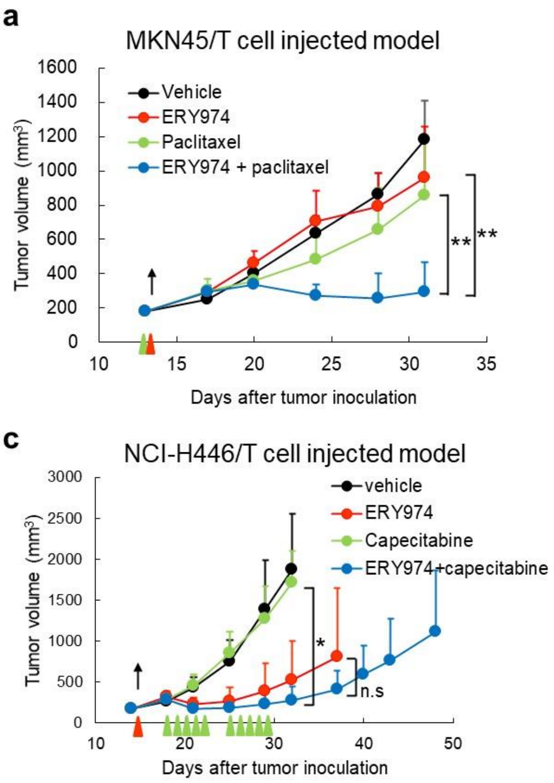

Supplementary Fig. 3. Combination of ERY974 and chemotherapy shows higher efficacy than ERY974 or chemotherapy alone in non-inflamed MKN45 and NCI-H446 tumours in T cell-injected models. a, ERY974 (1 mg/kg) + paclitaxel (20 mg/kg) efficacy in MKN45 xenograft tumours in the T cell-injected model (n = 5). **b,** ERY974 (1 mg/kg) + cisplatin (7.5 mg/kg) efficacy in MKN45 xenograft tumours in the T cell-injected model (n = 5). **c,** ERY974 (1 mg/kg) + capecitabine (540 mg/kg) efficacy in NCI-H446 xenograft tumours in the T cell-injected model (n = 5, one mouse in the capecitabine group was sacrificed due to toxicity on day 18, and one mouse in the combination group was sacrificed due to toxicity on day 25). The red and green arrows indicate the

timing of administration of ERY974 and each chemotherapy drug, respectively. The black arrow indicates the timing of T cell administration. Tumour volumes are presented as the mean ± SD. *$P < 0.05$, n.s., no significance (Wilcoxon chi-square test). In Supplementary Figure.3a, p value of ERY974 versus combination on day 31 is 0.00163, and p value of paclitaxel versus combination on day 31 is 0.0090. In Supplementary Figure.3b, p value of ERY974 versus combination, and p value of cisplatin versus combination on day 31 are both 0.0163. In Supplementary Figure.3c, p value of ERY974 versus combination on day 37 is 0.3272, and p value of capecitabine versus combination on day 32 is 0.0209.

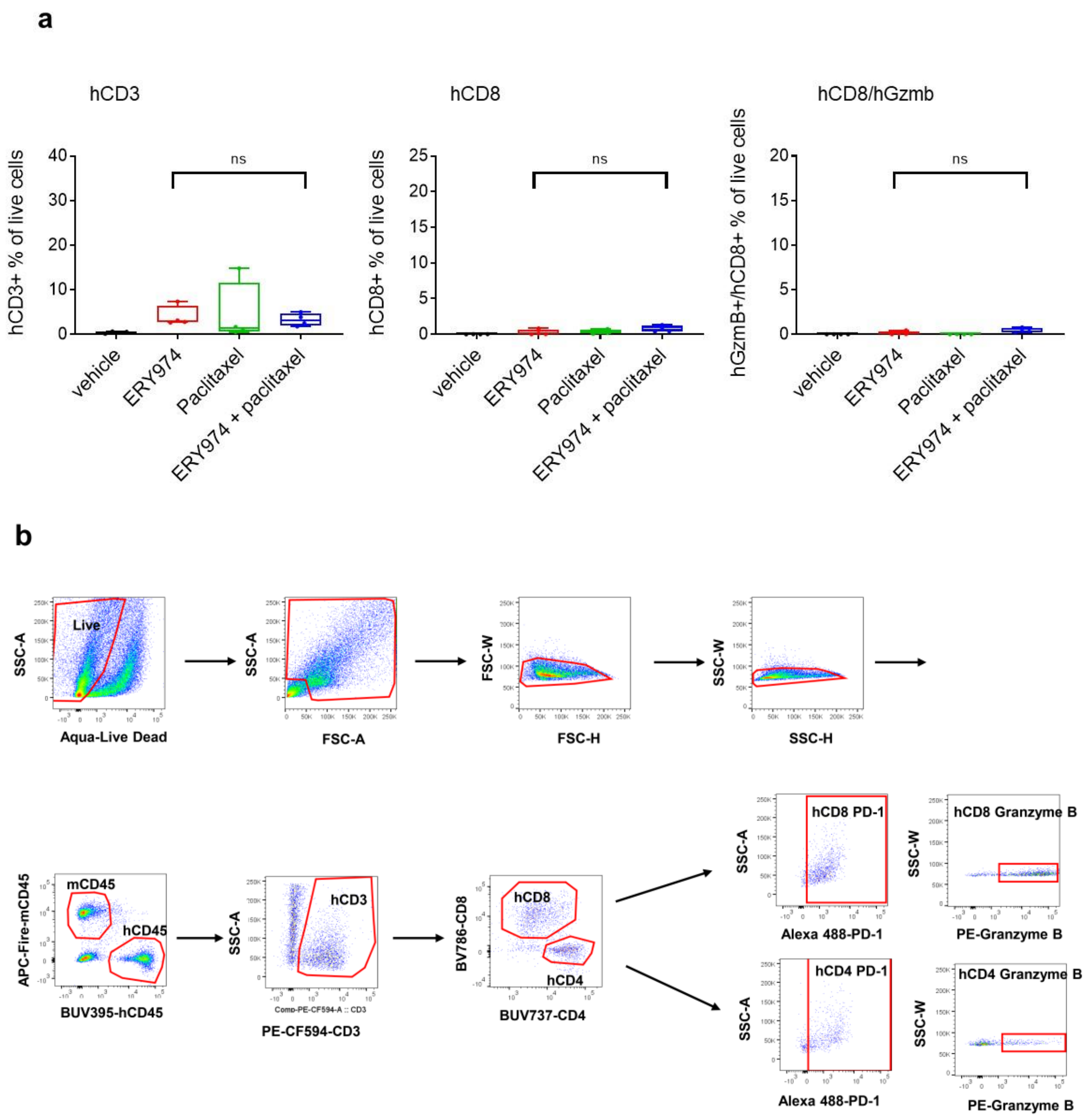

Supplementary Fig. 4. Infiltration and activation of T cells are not enhanced in the ERY974 + paclitaxel combination at early time point (day 2) in non-inflamed NCI-H446 tumours. a, Analysis of TILs isolated on day 2 from NCI-H446 tumours. When the tumour was established, 20 mg/kg paclitaxel was administered on day -1 and/or 5 mg/kg ERY974 was administered on day 0. NCI-H446 tumours were collected on day 2 (n = 4 per group). Data were

obtained using flow cytometry (n = 4). In the boxplot, centre lines show median values, box limits show upper and lower quartiles, and whiskers show minimum and maximum values. Statistical analysis was conducted by two-tailed unpaired *t*-test (n.s., not significant). **b,** Representative gating strategy of flow cytometry analysis.

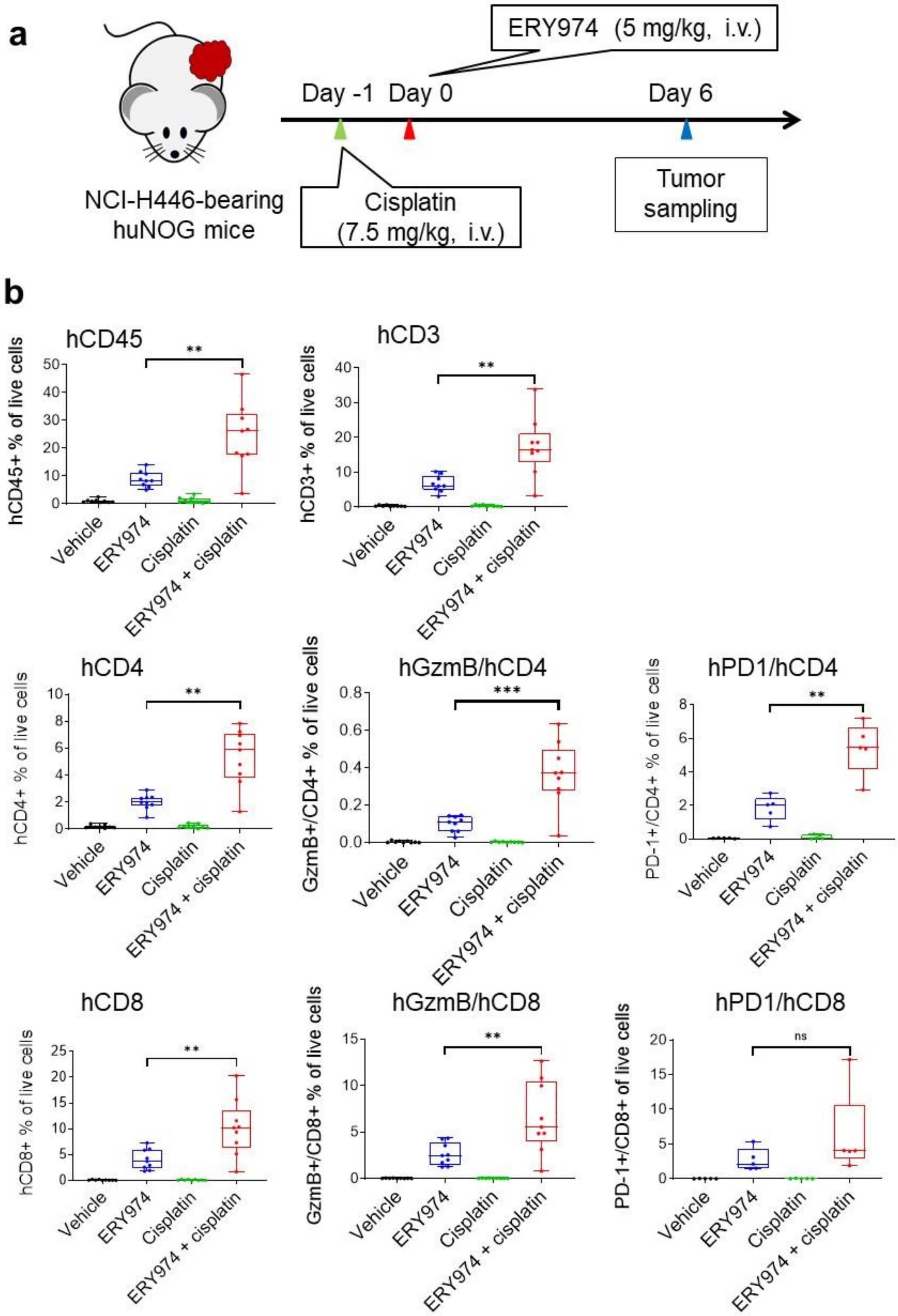
a
ERY974 (5 mg/kg, i.v.)
Day -1 Day 0 Day 6
NCI-H446-bearing huNOG mice
Cisplatin (7.5 mg/kg, i.v.)
Tumor sampling
b
hCD45
hCD45+ % of live cells
hCD3
hCD3+ % of live cells
hCD4
hCD4+ % of live cells
hGzmB/hCD4
GzmB+/CD4+ % of live cells
hPD1/hCD4
PD-1+/CD4+ % of live cells
hCD8
hCD8+ % of live cells
hGzmB/hCD8
GzmB+/CD8+ % of live cells
hPD1/hCD8
PD-1+/CD8+ of live cells
Vehicle
ERY974
Cisplatin
ERY974 + cisplatin
**
***
ns

c

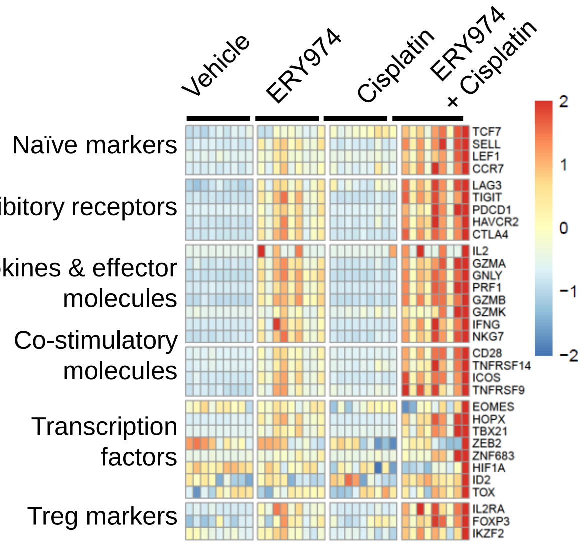
Vehicle
ERY974
Cisplatin
ERY974 + Cisplatin
Naïve markers
Inhibitory receptors
Cytokines & effector molecules
Co-stimulatory molecules
Transcription factors
Treg markers
TCF7
SELL
LEF1
CCR7
LAG3
TIGIT
PDCD1
HAVCR2
CTLA4
IL2
GZMA
GNLY
PRF1
GZMB
GZMK
IFNG
NKG7
CD28
TNFRSF14
ICOS
TNFRSF9
EOMES
HOPX
TBX21
ZEB2
ZNF683
HIF1A
ID2
TOX
IL2RA
FOXP3
IKZF2
2
1
0
−1
−2

d

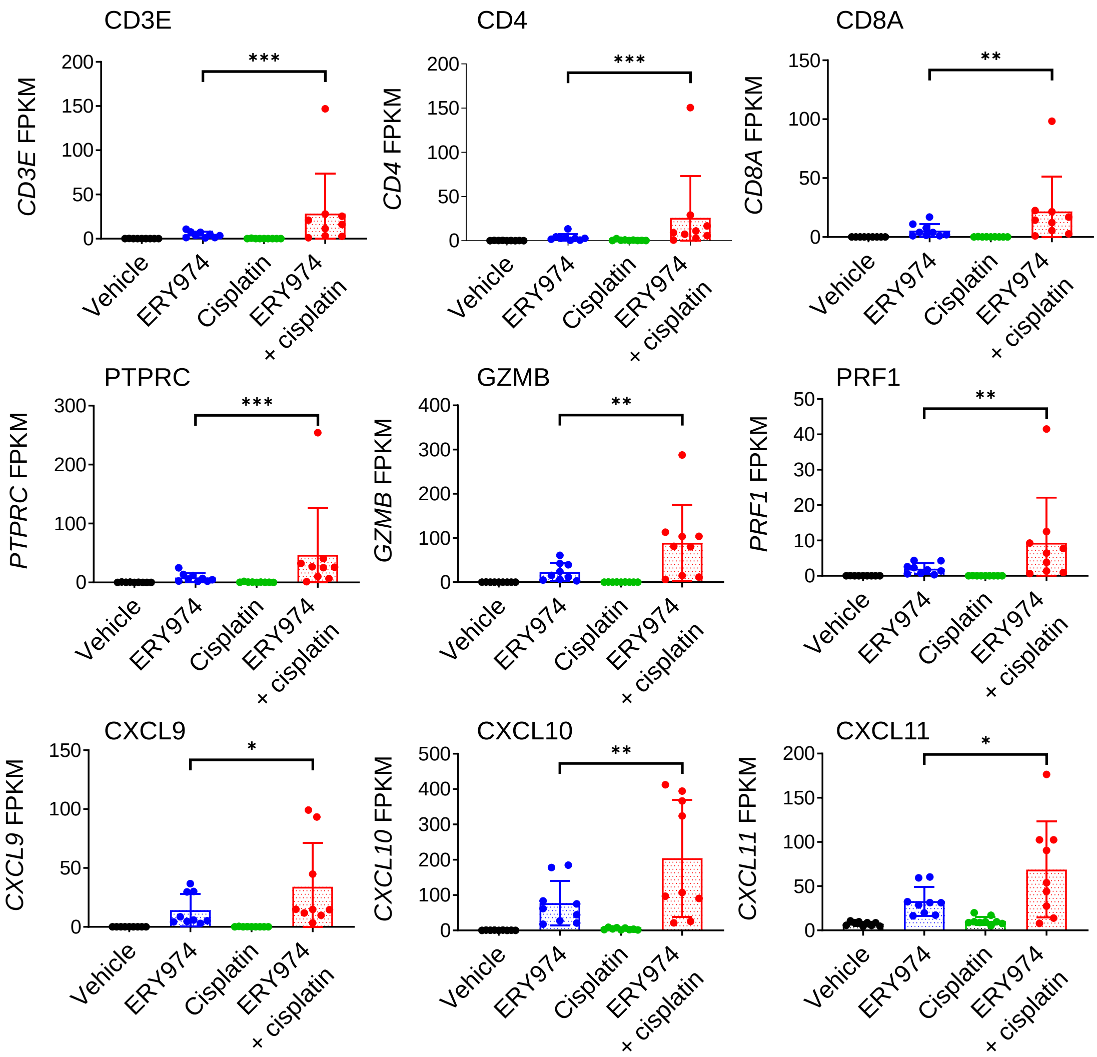
CD3E
CD3E FPKM
***
CD4
CD4 FPKM
***
CD8A
CD8A FPKM
**
PTPRC
PTPRC FPKM
***
GZMB
GZMB FPKM
**
PRF1
PRF1 FPKM
**
CXCL9
CXCL9 FPKM
*
CXCL10
CXCL10 FPKM
**
CXCL11
CXCL11 FPKM
*
Vehicle
ERY974
Cisplatin
ERY974 + cisplatin

Supplementary Fig. 5. **Infiltration and activation of T cells are enhanced in the ERY974 + cisplatin combination in non-inflamed NCI-H446 tumours. a,** Schematic of the experimental setup and tumour sampling schedule. NCI-H446 tumours collected on day 6 were used for TIL and RNA analyses (n = 9). Two independent experiments (n = 4 and 5) were combined. **b,** Analysis of TILs isolated on day 6 from NCI-H446 tumours using flow cytometry (n = 9 except for data of hPD-1+hCD4+ and hPD-1+hCD8+: n = 5). In the boxplot, centre lines show median values, box limits show upper and lower quartiles, and whiskers show minimum and maximum values. Statistical significance was determined by two-tailed unpaired *t*-test (*P < 0.05, **P < 0.01, ***P < 0.001, n.s., no significance). Exact p values of ERY974 versus combination in % of live cells for hCD45+, hCD3+, hCD4+, hGzmb+/hCD4+, hPD-1+/CD4+, hCD8+, hGzmb+/hCD8+, and hPD-1+/hCD8+ are 0.0069, 0.0037, 0.0054, 0.00030, 0.0079, 0.0090, 0.0094, and 0.1745, respectively. **c,** Heatmap of T cell signature genes using RNAseq data of day 6 samples (n = 9). The heatmap was created by calculating z-scores using log2-transformed FPKM values for all the target genes. **d,** mRNA levels of representative genes for T cell marker, T cell activation marker, cytokine, and chemokine using RNAseq data of day 6 samples. Data are

presented as the mean ± SD (n = 9). Statistical significance was conducted using edgeR glmQLFTest (*P < 0.05, **P < 0.01, ***P < 0.001 and n.s., no significance). Exact p values of ERY974 versus combination for *CD3E*, *CD4*, *CD8A*, *PTPRC*, *GZMB*, *PRF1*, *CXCL9*, *CXCL10*, and *CXCL11* are 0.000338, 0.000151, 0.00274, 0.000349, 0.00187, 0.00121, 0.0271, 0.00839, and 0.0108, respectively.

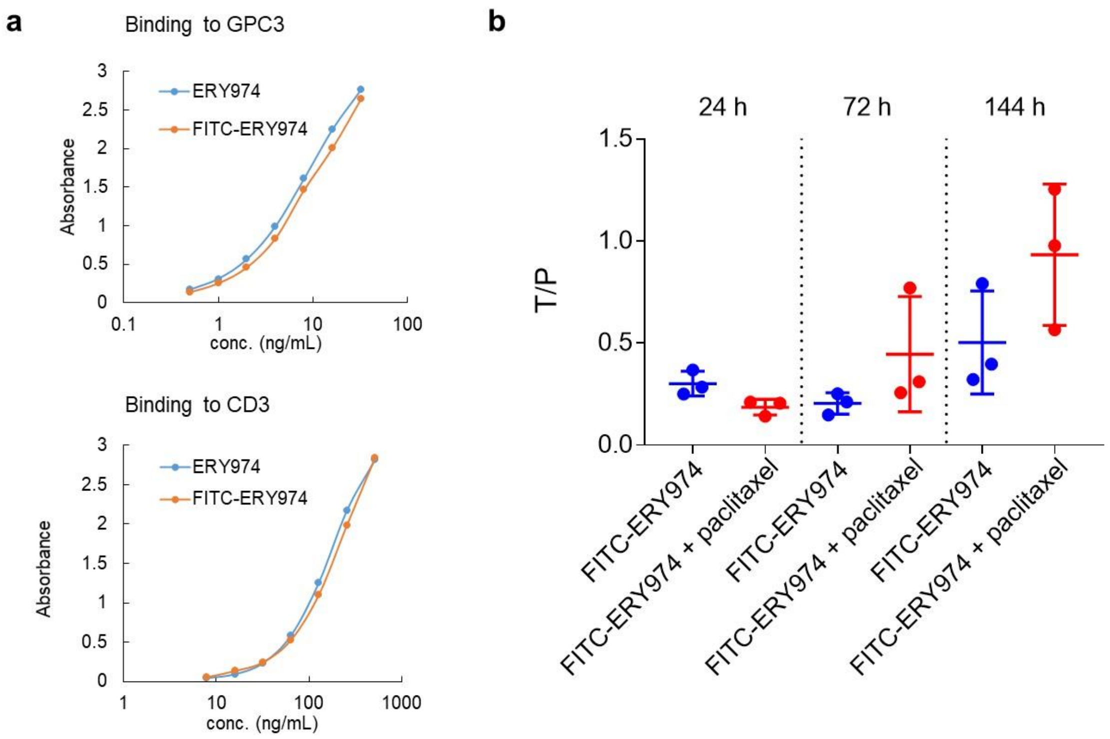

Supplementary Fig. 6. **Combination of FITC-labeled ERY974 with paclitaxel increases tumour to plasma ratio of FITC-labeled ERY974 in huNOG mice inoculated with NCI-H446. a,** Binding activity of non-labeled and FITC-labeled ERY974 to GPC3 and CD3 by ELISA (n = 1). **b,** Tumour (T) to plasma (P) ratio of FITC-labeled ERY974 (n = 3). Data are presented as the mean ± SD.

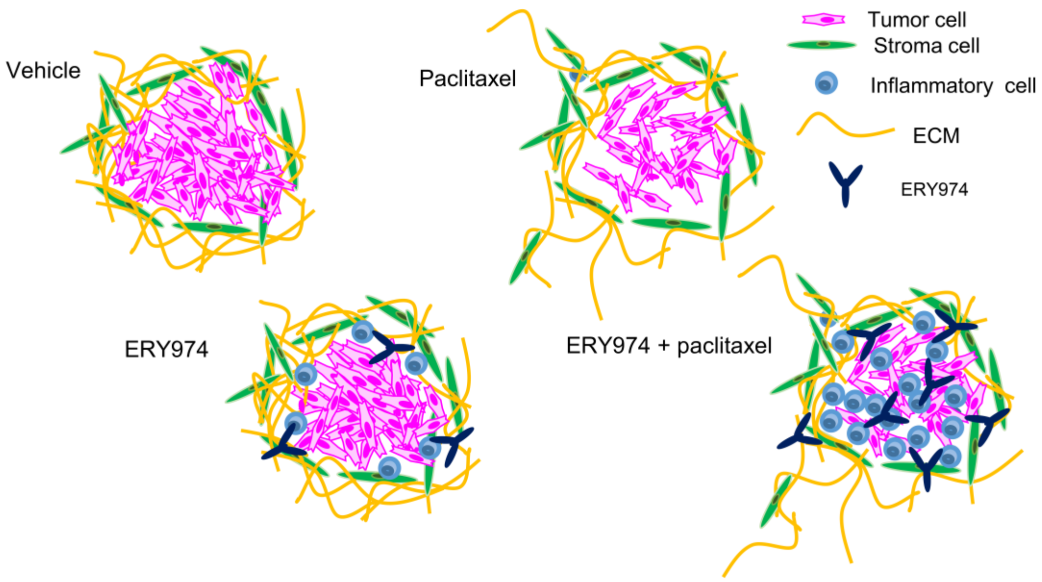

Supplementary Fig. 7. Model illustrating the mechanism underlying the synergistic effect of ERY974 and paclitaxel. ERY974 monotherapy leads to T cell infiltration into the tumour, although T cells and ERY974 remain at the tumour-stromal boundary, as the tumour structure is tightly packed. However, when combined with paclitaxel, the tumour structure is disrupted, allowing T cells and ERY974 to infiltrate into the tumour centre, thus improving antitumour efficacy.

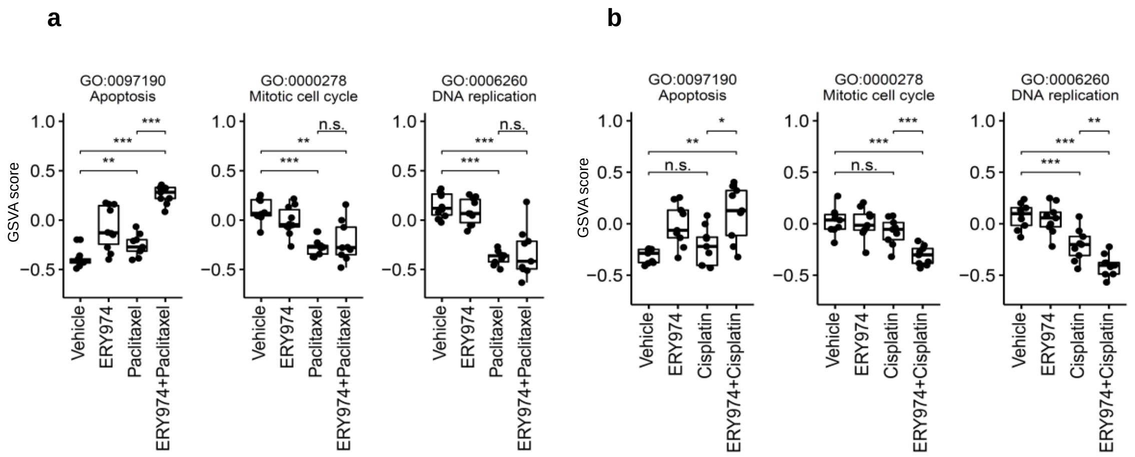

Supplementary Fig. 8. ERY974 enhances paclitaxel- or cisplatin-induced inhibition of mitosis. Enrichment score of gene ontology (GO) for apoptosis, mitotic cell cycle, and DNA replication. **a,** ERY974 + paclitaxel combination (n = 9 for paclitaxel and combination groups, n = 10 for vehicle and ERY974 groups). In the boxplot, centre lines show median values, box limits show upper and lower quartiles, and whiskers show minimum and maximum values within quartiles ± 1.5 IQR (interquartile range). *P < 0.05, **P < 0.01, ***P < 0.001, n.s., not significant (two-tailed unpaired *t*-test). Exact p values of vehicle versus paclitaxel, vehicle versus combination, and paclitaxel versus combination for apoptosis GO are 5.49×10^{-3}, 1.73×10^{-11}, 1.64×10^{-8}, respectively. Exact p values of vehicle versus paclitaxel, vehicle versus combination, and paclitaxel versus combination for mitotic cell cycle GO are 4.09×10^{-7}, 1.24×10^{-3}, 4.34×10^{-1}, respectively. Exact p values of vehicle versus paclitaxel, vehicle versus

combination, and paclitaxel versus combination for DNA replication GO are 1.20 $\times 10^{-8}$, 2.84 $\times 10^{-4}$, 5.52 $\times 10^{-1}$, respectively. **b,** ERY974 + cisplatin combination (n = 9). In the boxplot, centre lines show median values, box limits show upper and lower quartiles, and whiskers show minimum and maximum values within quartiles ± 1.5 IQR (interquartile range). *P < 0.05, **P < 0.01, ***P < 0.001, n.s., not significant (two-tailed unpaired *t*-test). Exact p values of vehicle versus cisplatin, vehicle versus combination, and cisplatin versus combination for apoptosis GO are 1.55 $\times 10^{-1}$, 1.34 $\times 10^{-3}$, 1.01 $\times 10^{-2}$, respectively. Exact p values of vehicle versus cisplatin, vehicle versus combination, and cisplatin versus combination for mitotic cell cycle GO are 1.11 $\times 10^{-1}$, 1.56 $\times 10^{-5}$, 6.46 $\times 10^{-4}$, respectively. Exact p values of vehicle versus cisplatin, vehicle versus combination, and cisplatin versus combination for DNA replication GO are 6.78 $\times 10^{-4}$, 1.97 $\times 10^{-7}$, 5.77 $\times 10^{-3}$, respectively.

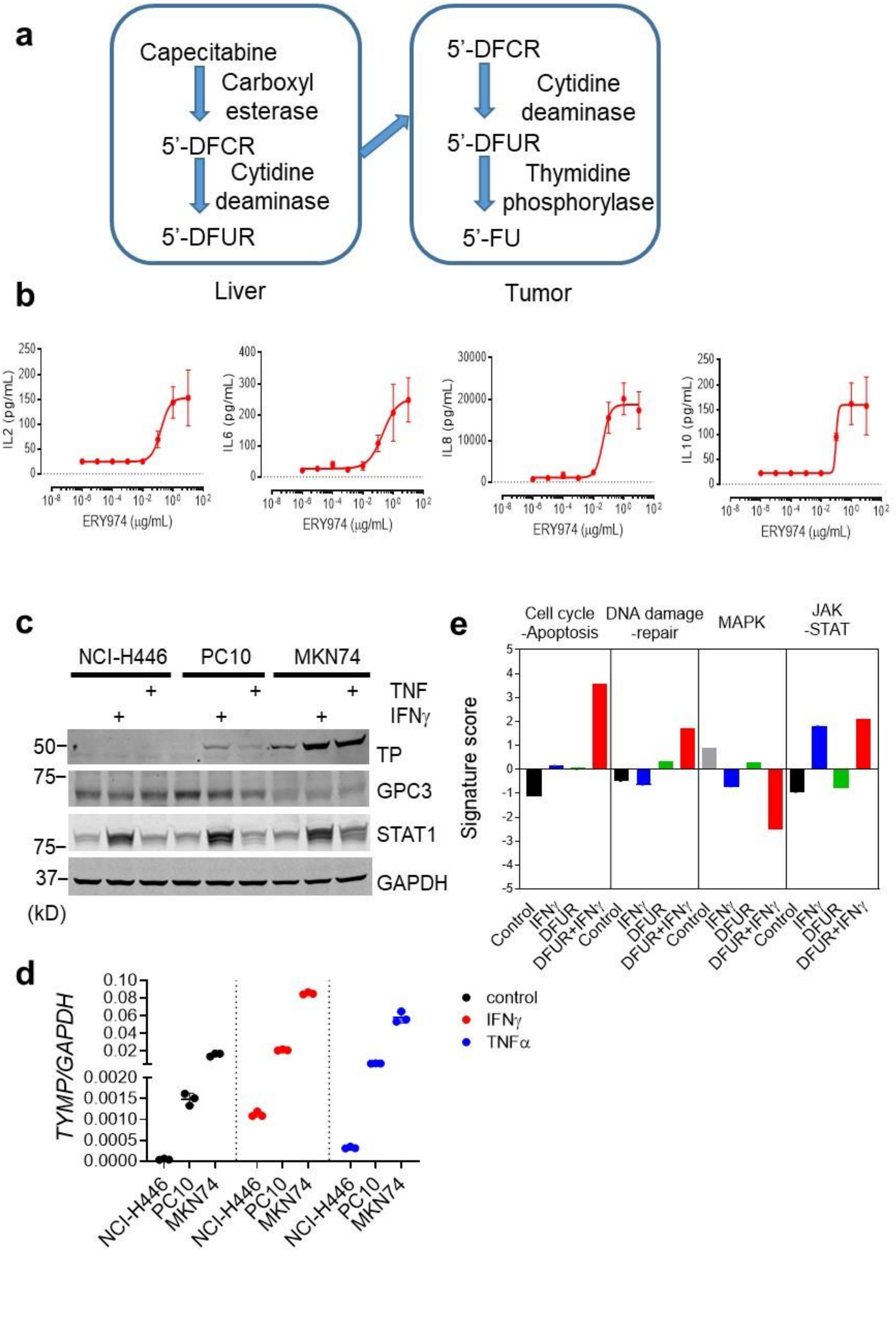
a
Capecitabine
Carboxyl esterase
5'-DFCR
Cytidine deaminase
5'-DFUR
Liver
5'-DFCR
Cytidine deaminase
5'-DFUR
Thymidine phosphorylase
5'-FU
Tumor
b
IL2 (pg/mL)
IL6 (pg/mL)
IL8 (pg/mL)
IL10 (pg/mL)
ERY974 (μg/mL)
c
NCI-H446
PC10
MKN74
TNF
IFNγ
TP
GPC3
STAT1
GAPDH
(kD)
d
TYMP/GAPDH
NCI-H446
PC10
MKN74
control
IFNγ
TNFα
e
Cell cycle -Apoptosis
DNA damage -repair
MAPK
JAK -STAT
Signature score
Control
IFNγ
DFUR
DFUR+IFNγ

Supplementary Fig. 9. **IFNγ and TNFα induces TP, and sensitises MKN45 to 5'-DFUR, a 5'-FU precursor. a,** Metabolic conversion of capecitabine to its active form, 5'-FU. **b,** Cytokine production (IL2, IL6, IL8, and IL10) in culture medium of TDCC (n = 3) measured by MAGPIX. Data are presented as the mean ± SD. **c**, Western blot analysis for TP expression in NCI-H446, PC10, and MKN74 cells after treatment with recombinant IFNγ and TNFα at 100 ng/mL for 24 h (n = 1). **d,** qRT-PCR analysis for *TYMP* gene expression in NCI-H446, PC10, and MKN74 cells after treatment with recombinant IFNγ and TNFα at 100 ng/mL for 24 h (n = 3). *TYMP* (TP) expression is normalised by *GAPDH* expression. Data are presented as the mean. **e,** Pathway scoring analysis using nSolver pathway scoring module and nCounter data.

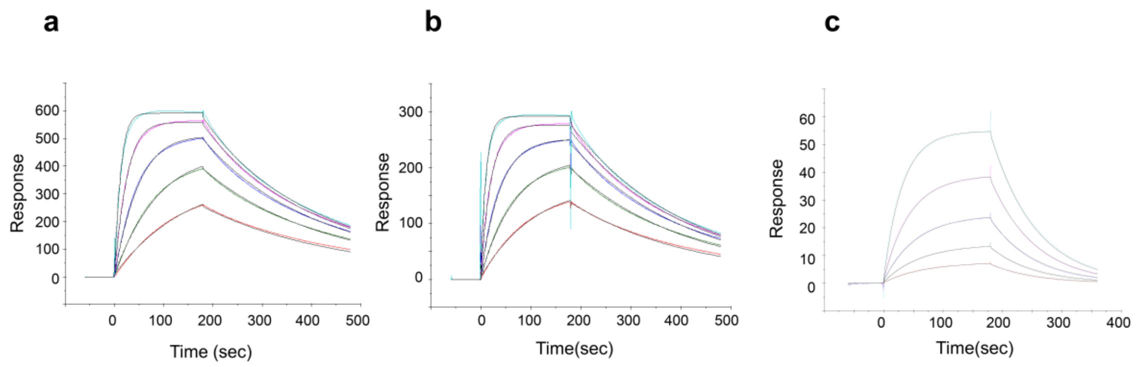

Supplementary Fig. 10. **SPR sensorgrams of EGFR-TRAB. a,** Binding of cetuximab to 6.25 nM (red), 12.5 nM (green), 25 nM (purple), 50 nM (pink), and 100 nM (blue) of EGFR (n = 1). **b,** Binding of EGFR-TRAB to 6.25 nM (red), 12.5 nM (green), 25 nM (purple), 50 nM (pink), and 100 nM (blue) of EGFR (n = 1). **c.** Binding of EGFR-TRAB to 75 nM (red), 150 nM (green), 300 nM (purple), 600 nM (pink), and 1200 nM (blue) of CD3εγ (n = 1).

Supplementary Table 1. List of antibodies used for flow cytometric analysis.

Label	Target antigen	Ab clone	Company	Cat. no.	Dilution
BV395	hCD45	HI30	BD Biosciences (Franklin Lakes, NJ, USA)	563792	1:50
PE-CF594	CD3	UCHT1	BioLegend	300450	1:50
BUV737	CD4	SK3	BD Biosciences	612748	1:50
BV786	CD8	RPA-T8	BioLegend	301046	1:50
Alexa488	PD-1	EH12.2H7	BioLegend	329936	1:50
PE	GZB	GB11	BD Biosciences	561142	1:50
